# *In Vivo* Time-Lapse Imaging Reveals Differential Activity-Induced Regulation of Proteasome Activity in Subcellular Regions of the Optic Tectum in *Xenopus laevis* Tadpoles

**DOI:** 10.1021/acschemneuro.5c00881

**Published:** 2026-04-14

**Authors:** Jessica M. Lin, Benjamin Baah Konadu, Darci J. Trader, Hai-yan He

**Affiliations:** † Biology Department, 8368 Georgetown University, Washington, D.C. 20057, United States; ‡ Department of Pharmaceutical Sciences, 8788 University of California, Irvine, California 92697, United States

**Keywords:** : proteasome activity, substrate-based probe, in vivo time-lapse imaging, subcellular compartment, neuronal activity, optic tectum

## Abstract

The proteasome is a major organelle responsible for protein degradation in neurons and has been implicated in the regulation of signal transduction and activity-dependent plasticity mechanisms that are essential for normal neuronal function. However, our understanding of the regulation of proteasome activity in the brain is limited by the currently available assays and tools. Here, we used a fluorogenic substrate-based probe, TAS1, to directly monitor proteasome activity in the brain of *Xenopus laevis* tadpoles with time-lapse two-photon microscopy. With the spatial resolution enabled by *in vivo* imaging, our data revealed a significant difference in proteasome activity between brain regions enriched in neuronal soma versus neuropil under both basal and pharmacologically stimulated conditions, suggesting differential activity-induced regulation of proteasome activity across neuronal subcellular compartments. These results demonstrate the feasibility of using TAS1 to track proteasome activity *in vivo* and provide new evidence for the differential regulation of proteasome activity in different subcellular compartments of neurons in the intact neural circuit.

## Graphical abstract

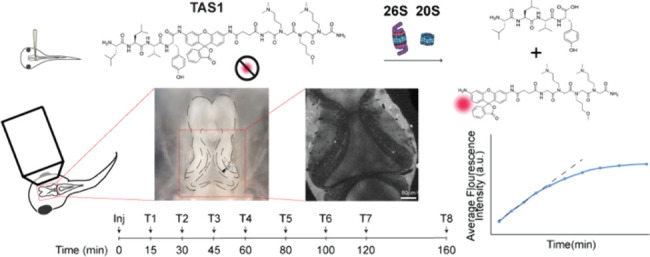

Protein degradation is critical for maintaining protein homeostasis and eliminating misfolded, damaged, aggregated, or other unwanted proteins. In neurons, one major organelle responsible for protein degradation is the proteasome, which is estimated to mediate >60% of protein degradation under physiological conditions.^1^ Proteasomes consist of a 20S core particle and, in some cases, one or two 19S regulatory caps. The 20S core particle along with the 19S cap is known as the 26S proteasome and degrades proteins through ubiquitin-dependent pathways.^1,2^ Emerging evidence suggests that stand-alone 20S core particles can also function independently to degrade non-ubiquitinated proteins.^3–7^ The functional roles proteasomes play in the nervous system are multifaceted. In addition to degrading dysfunctional proteins, proteasomes are also involved in the regulation of neuronal transmission as well as activity-dependent plasticity mechanisms.^8–11^ Conversely, proteasome activity has also been demonstrated to be regulated in response to neuronal activity in primary neuronal cultures,^12–14^ as well as following fear conditioning in tissue lysates of mouse amygdala and hippocampus.^15,16^ Proteasomes are found in different subcellular compartments in neurons, where their activity may be differentially regulated to respond to localized proteostatic demands.^11^ For instance, upon synaptic stimulation, proteasomes are sequestered into dendritic spines in an *N*-methyl-d-aspartate (NMDA) receptor and calcium/calmodulin-dependent protein kinase II (CaMKIIα) dependent manner, where they regulate synaptic plasticity.^17–19^

Current tools to measure proteasome activity include fluorogenic substrates that are used in *in vitro* assays with tissue lysate or cell culture,^20,21^ activity-based probes that irreversibly bind to and label active proteasomes,^22–25^ and ubiquitin-fusion degradation substrates such as Ub^G76V^-GFP that quantify ubiquitin–proteasome-dependent proteolysis.^26^ These tools have enabled quantitative evaluation of proteasome activity in neuronal tissue and contributed substantially to our current understanding of the regulation of proteasome activity and their involvement in various neuronal functions. However, due to the complexity of neuronal morphology and functions, methods allowing real-time tracking of proteasome activity in live animals under physiological conditions are needed to better understand spatiotemporal regulation of proteasome activity *in vivo*. *In vitro* assays using fluorogenic substrates are limited in their ability to detect subcellular distribution of proteasome activity, while activity-based probes can be used for live cell imaging but only provide snapshots of proteasome activity due to irreversible binding of the probes to active proteasomes.^27^ The use of Ub^G76V^-GFP allows for relatively rapid quantification of ubiquitin–proteasome-dependent proteolytic activity in live cells and *in vivo* using a transgenic mouse model.^26,28^ However, the need for genetic manipulation to introduce the exogenously expressed Ub^G76V^-GFP reporter limits its application, and the fluorescence readout is confounded by the synthesis rate of the reporter. Furthermore, this method is not able to detect the ubiquitin-independent activity of standalone 20S proteasomes.

To address these limitations, we used the TAS1 probe *in vivo* in albino *Xenopus laevis* tadpoles to track proteasome activity levels across different brain regions in real time with time-lapse imaging. TAS1 is a fluorogenic substrate-based probe consisting of rhodamine 110 (Rh110) conjugated to a Leu-Leu-Val-Tyr (LLVY) peptide on one side and a short peptoid fragment on the other side for greater cell permeability (Figure 1A). Compared to the commonly used fluorogenic substrate Succinyl-Leu-Leu-Val-Tyr-7-amido-4-methylcoumarin (Suc-LLVY-AMC), TAS1 shows significantly higher fluorescent intensity upon proteasome cleavage.^21^ Furthermore, the excitation (485 nm) and emission (535 nm) of TAS1 fall in the visible light range, making it a more preferable choice for *in vivo* imaging, unlike the Suc-LLVY-AMC probe, which has an excitation and emission in the ultraviolet range and thus can induce phototoxicity in live tissue (unpublished observation). Intraventricular injections in the tadpole brain enable rapid diffusion of injected reagents into the surrounding brain regions, allowing for high spatial and temporal control. We examined proteasome activity in the optic tectum, which is the major visual processing center in tadpole brains and has well-characterized cytoarchitecture and functional connectivity.^29^ We showed that the TAS1 probe can be used to quantitatively evaluate proteasome activity *in vivo*. The increase in TAS1 fluorescence signal over time is significantly inhibited by proteasome inhibitors epoxomicin and MG-132. Conversely, increasing neuronal activity by pharmacological stimulation significantly increased the level of proteasome activity in the brain as measured by TAS1 *in vivo*. This activity-induced increase in the overall proteasome activity was confirmed with Suc-LLVY-AMC-based *in vitro* assays using brain lysates. In contrast to the in vitro assay, the TAS1-based *in vivo* assay allowed us to compare the proteasome activity in different brain regions within the optic tectum. Interestingly, we observed a significantly higher level of proteasome activity in the neuropil region, which contains mostly neuronal processes, than in the cell body layer, which contains mostly neuronal soma. Following pharmacological stimulation, the neuronal-activity-induced increase in proteasome activity was also more prominent in the neuropil region, suggesting differential subcellular regulation of proteasome activity. These results demonstrate that TAS1-based *in vivo* imaging can be used as an effective tool to monitor proteasome activity across different brain regions and subcellular compartments in live tissue.

**Figure 1:**
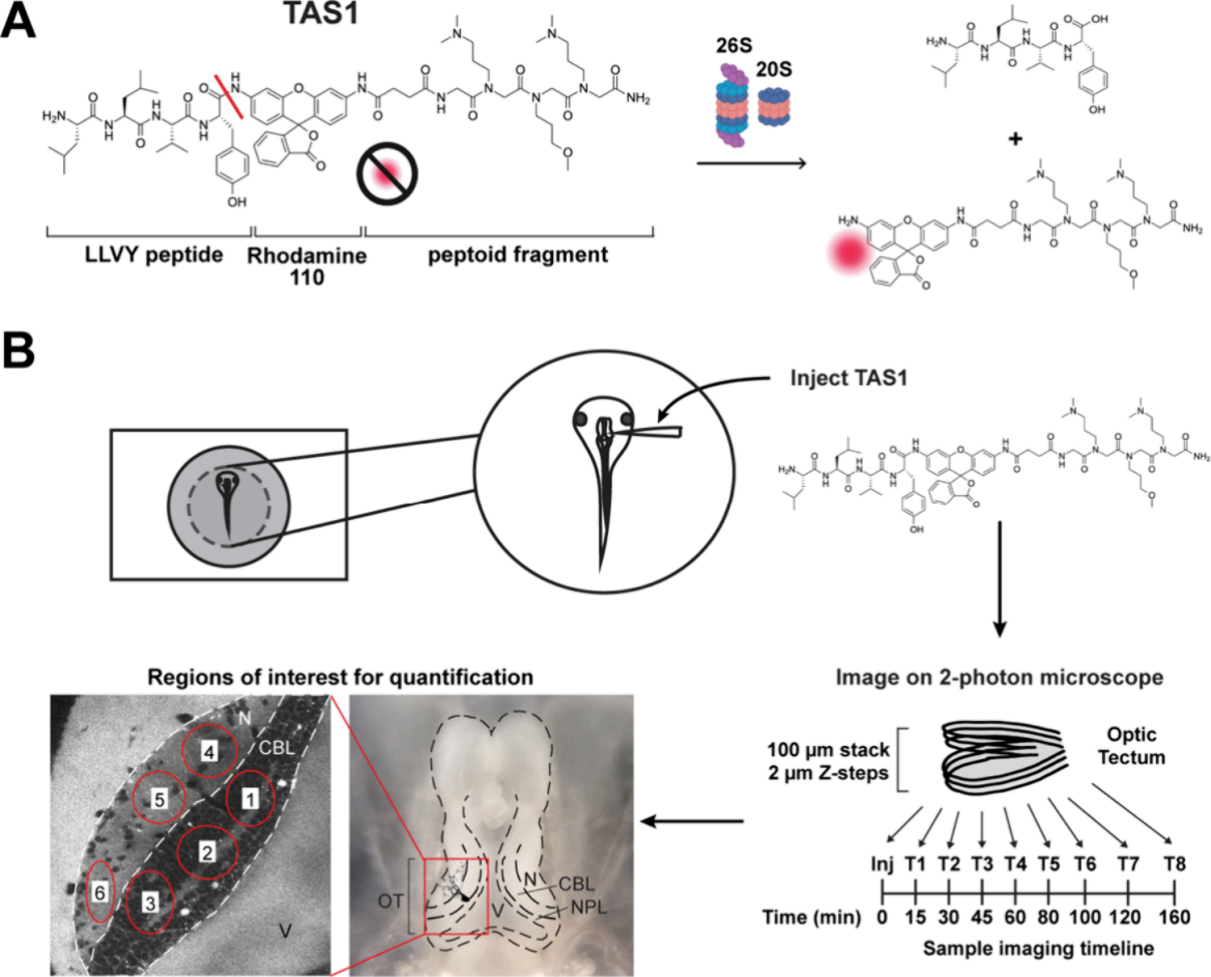
Experimental setup and analysis methods. (A) Chemical structure of the TAS1 probe and its cleaved products. (B) Schematic of the experimental setup. Awake tadpole was immobilized in the imaging chamber, and TAS1 was injected intraventricularly. The animal was then imaged on a 2-photon microscope. Z-stacks of one tectal lobe were acquired at different time points with the same acquisition parameters. Regions of interest (ROIs) were drawn for quantification, and average intensity in each ROI was determined at every time point. V: ventricle, N: neuropil, CBL: cell body layer; NPL: neural progenitor layer. ROIs were drawn across the cell body layer (ROIs 1–3) and neuropil (ROIs 4–6) regions.

To effectively deliver TAS1 to the optic tectum, we injected TAS1 into the midbrain ventricle of live tadpoles and imaged one lobe of the optic tectum every 10–15 minutes for up to 3 hours with *in vivo* two-photon microscopy (Figure 1B). We then quantified the average fluorescent intensity of the cleaved probes accumulated over time as a proxy for proteasome activity. The optic tectum can be roughly divided into two main regions: the cell body layer, which is packed with neuronal soma, and the neuropil, which contains mostly axons and dendrites with sparsely distributed neuronal soma. This unique anatomical structure gives us the opportunity to examine proteasome activity in subcellular compartments in the brain. To determine whether the cell body layer and neuropil exhibit different levels of proteasome activity, we analyzed the fluorescence in these two regions using separate regions of interest (Figure 1B, also see the Materials and Methods section for details).

We first tested whether TAS1 is cleaved in the tadpole brain under the basal condition. Fluorescent signal from the TAS1 probe was detected in the brain within minutes of injection (Figure 2A). The signal increased over time and could be seen in both the cell body layer and the neuropil layer (Figure 2B), suggesting gradual degradation of the probe by proteasomes *in vivo*. The diffusion and uptake of the probe were relatively even along both medial-lateral and rostral-caudal axes in both tectal lobes (Figure 2A,B, also see Supplemental Video). Higher magnification images showed that the TAS1 signal was most visible in the cytoplasm, including in the cytoplasm around the nuclei in the cell body layer, as well as in neuronal processes in the neuropil layer (Figure 2C). Interestingly, despite the abundance of proteasome subunits expressed in the nuclei in neurons,^30,31^ TAS1 signals in the nuclei were relatively low in most cells, with the exception of a small subset of cells that showed high TAS1 fluorescent intensity in the nuclei (Figure 2C). The relatively lower level of proteasome activity in the nuclei compared to cytoplasm has also been reported in pig brain slice.^32^ Additionally, we observed strong TAS1 signals in the ventricle and the subarachnoid space surrounding the brain (Figure 2B,C), which could be due to cleavage by extracellular proteasomes in the cerebral spinal fluid.^33,34^ The TAS1 probe does not penetrate the blood brain barrier in healthy animals, which allowed us to use blood vessels as reliable landmarks for image alignment during imaging acquisition and processing (Figure 2D).

**Figure 2:**
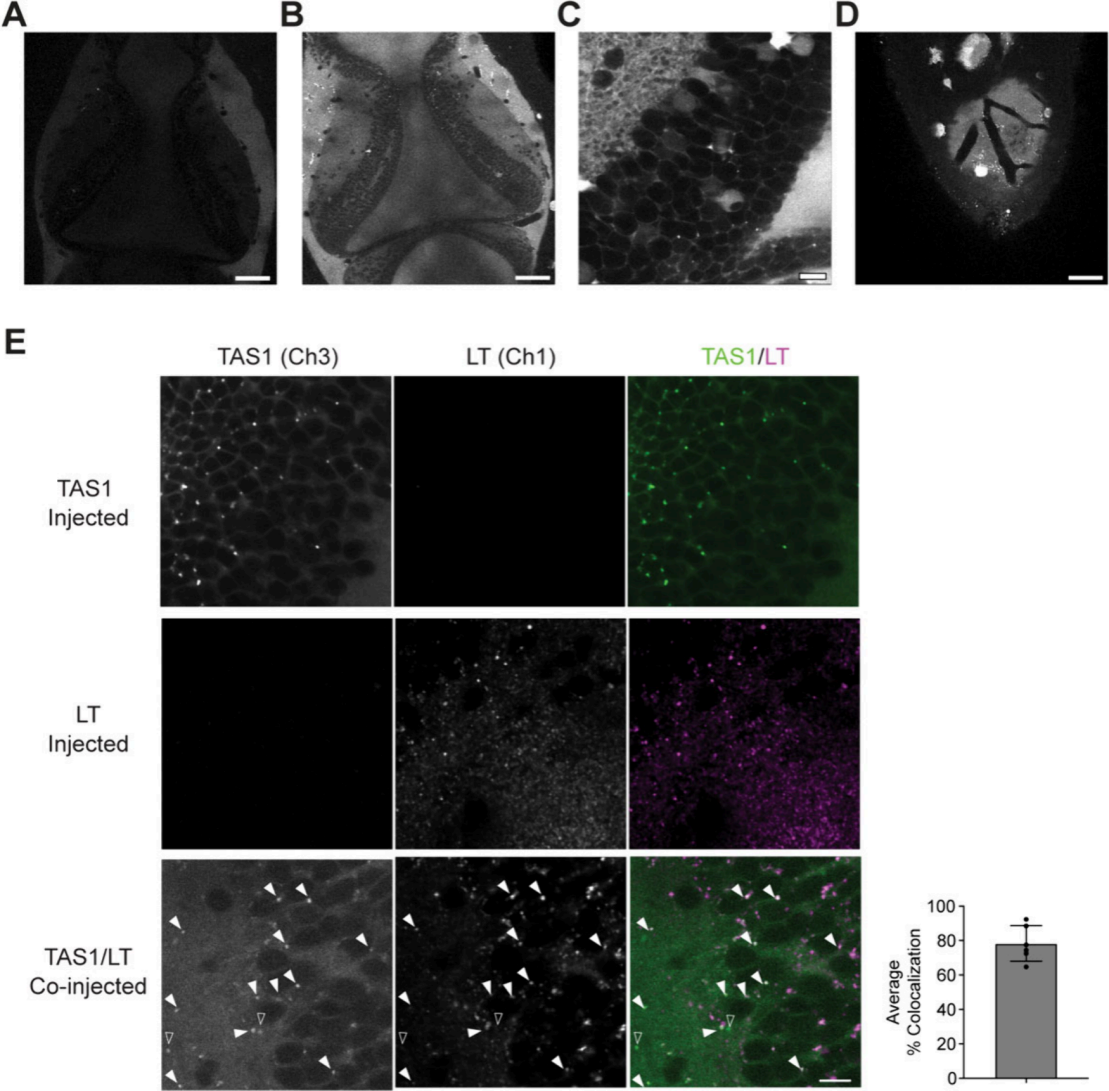
TAS1 fluorescence signal can be detected in live tadpole brains. (A, B) Representative image of TAS1 fluorescent signal in the optic tectum 10 min (A) and 45 min (B) following intraventricular injection. Tectal lobes shown 120–140 μm from the dorsal surface. (C) Zoomed in image of TAS1 signal in one tectal lobe 30 min post-injection. (D) Dorsal surface of the brain where blood vessels can be seen. (E) TAS1 puncta colocalize with LysoTracker Red puncta *in vivo*. Representative images are shown for animals injected with TAS1 only, LysoTracker Red (LT) only, or coinjected with both TAS1 and LT. Histogram on the right shows the average percentage of TAS1 puncta that colocalized with LT puncta across all images (mean ± SEM, *n* = 6). Scale bar: A, B, D, 80 μm; C, E, 10 μm. All images in this and the following figures are single optical sections.

We also observed puncta of the TAS1 signal in some cells, particularly at later time points of the imaging session (Figure 2C, also see Figure 3A). Previous work using TBZ1, a fluorogenic probe for the immunoproteasome that differs from TAS1 only by its peptide recognition sequence (Ala-Thr-Met-Trp in TBZ1 versus Leu-Leu-Val-Tyr in TAS1) reported localization of TBZ1 signal in acidic organelles, which include the endosome and lysosome.^35^ To test whether the puncta resulted from degraded TAS1 probes similarly localized to acidic organelles, we coinjected LysoTracker Red (LT) with TAS1. As expected, LysoTracker Red puncta were seen in the cytoplasm, both in the cell body layer and in the neuropil, but were not seen in the nuclei (Figure 2E). Quantification showed that the TAS1 puncta mostly colocalized with LysoTracker Red puncta (percent colocalization: 78.31 ± 3.56, Mean ± SEM, Figure 2E).

**Figure 3:**
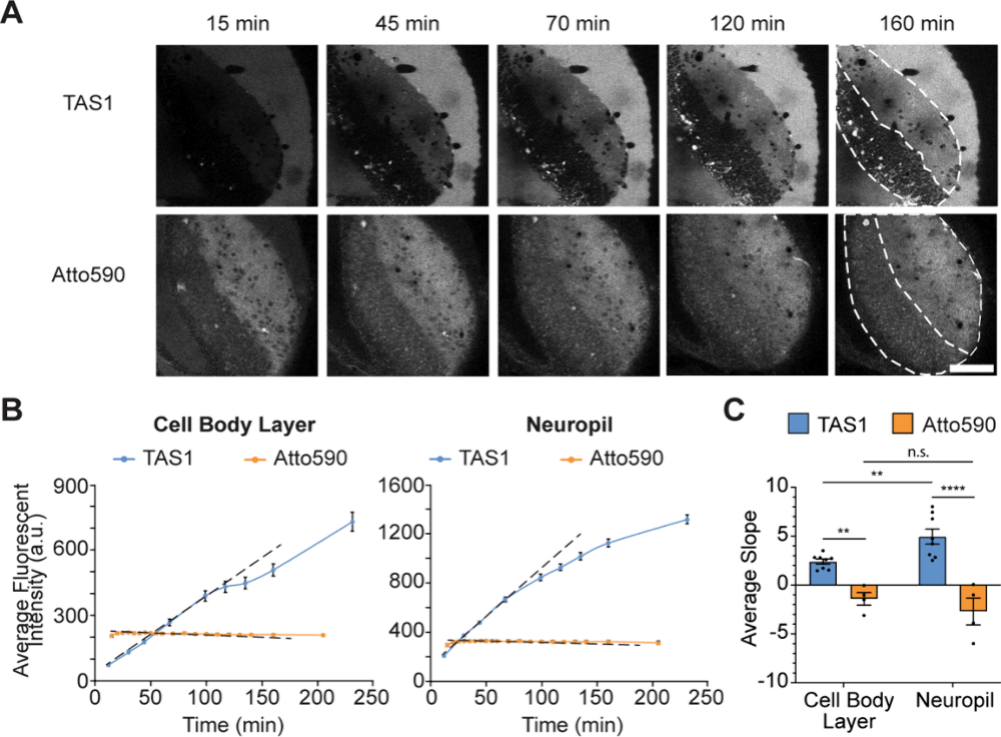
TAS1 signal increases over time in live tadpole brains. (A) Representative time-lapse images of TAS1 and Atto590 signals in the optic tectum. (B) Quantification of average fluorescence intensity over time in the cell body layer (CBL) and neuropil layer (N) in the representative animals. The linear range of each curve was used to calculate the slope (dashed line). (C) Average slopes in CBL and N across animals injected with TAS1 or Atto590. TAS1: *n* = 9, Atto590: *n* = 4; **: *p* < 0.01, ****: *p* < 0.0001, n.s.: not significant, two-way ANOVA with Holm–Šídák correction for multiple comparisons. Scale bar: 80 μm.

As with other substrate-based proteasome activity probes, the increase in the fluorescent intensity over time can be used as a measurement of the level of proteasome activity in the tissue. We took measurements of the average intensity of TAS1 signal in the cell body layer and neuropil layer of the tectal lobe at different time points following intraventricular injection of the probe (Figure 3A). In both regions, TAS1 fluorescence increased linearly for the first 60–90 minutes following TAS1 injection and then slowed down before eventually reaching a plateau (Figure 3B). To account for possible animal-to-animal variabilities in the exact timing of imaging time points in reference to the intraventricular injection, instead of taking the absolute fluorescence intensity at a certain time point, we calculated the slope of the linear range of the TAS1 fluorescent intensity curve for each animal as a proxy for the overall proteasome activity level. Interestingly, we observed significantly higher slopes in the neuropil than in the cell layer, indicating significantly higher levels of proteasome activity in the neuropil (Figure 2C). To control for the temporal dynamics of the initial diffusion and uptake of the probe into the brain tissue as well as its clearance, we intraventricularly injected a fluorescent dye, Atto590, and performed time-lapse *in vivo* imaging with the same time intervals and duration. Similar to TAS1, the Atto590 signal was detected in both the cell body layer and neuropil within minutes of injection (Figure 3A). However, in contrast to the TAS1 signal, the Atto590 signal showed no further increase throughout the imaging session but instead decreased slightly over time (Figure 3B). This confirms that the diffusion and uptake of such small molecule probes and dyes in the tadpole brain tissue is rather fast, and the accumulation of the TAS1 signal we observed in brain cells likely resulted from the cleavage of the probes rather than gradual diffusion of the probes into the tectal lobe. As expected, the slopes for Atto590 were significantly lower than those of TAS1 and were mostly in the negative range, consistent with the gradual decay of the signal observed over time (Figure 3C). Overall, these data demonstrate that the TAS1 probe is cleaved at detectable levels *in vivo* under the basal condition.

To validate that the TAS1 fluorescent signal resulted from cleavage by proteasomes in the brain, we coinjected proteasome inhibitors epoxomicin (EP) and MG-132 with TAS1 and compared the accumulation of TAS1 fluorescence over time in these animals to that of animals injected with only TAS1.^36,37^ The presence of proteasome inhibitors resulted in a drastic reduction in the average TAS1 fluorescence intensity in the tadpole brain across the cell body layer and the neuropil layer (Figure 4A,B). Accordingly, the slopes of fluorescence accumulation were also significantly reduced in animals treated with EP and MG-132 compared to batch-matched controls injected with TAS1 only, suggesting that EP and MG-132 effectively inhibited TAS1-measured proteolytic activity in the tadpole brain (Figure 4C). The reduction of the fluorescent signal was also observed in the ventricle, confirming that the signals seen in the ventricle resulted from proteasome activity (Figure 4A). Taken together, these data confirm that proteasome-mediated cleavage of the TAS1 probes is the primary source of the accumulated fluorescence detected in the brain.

**Figure 4:**
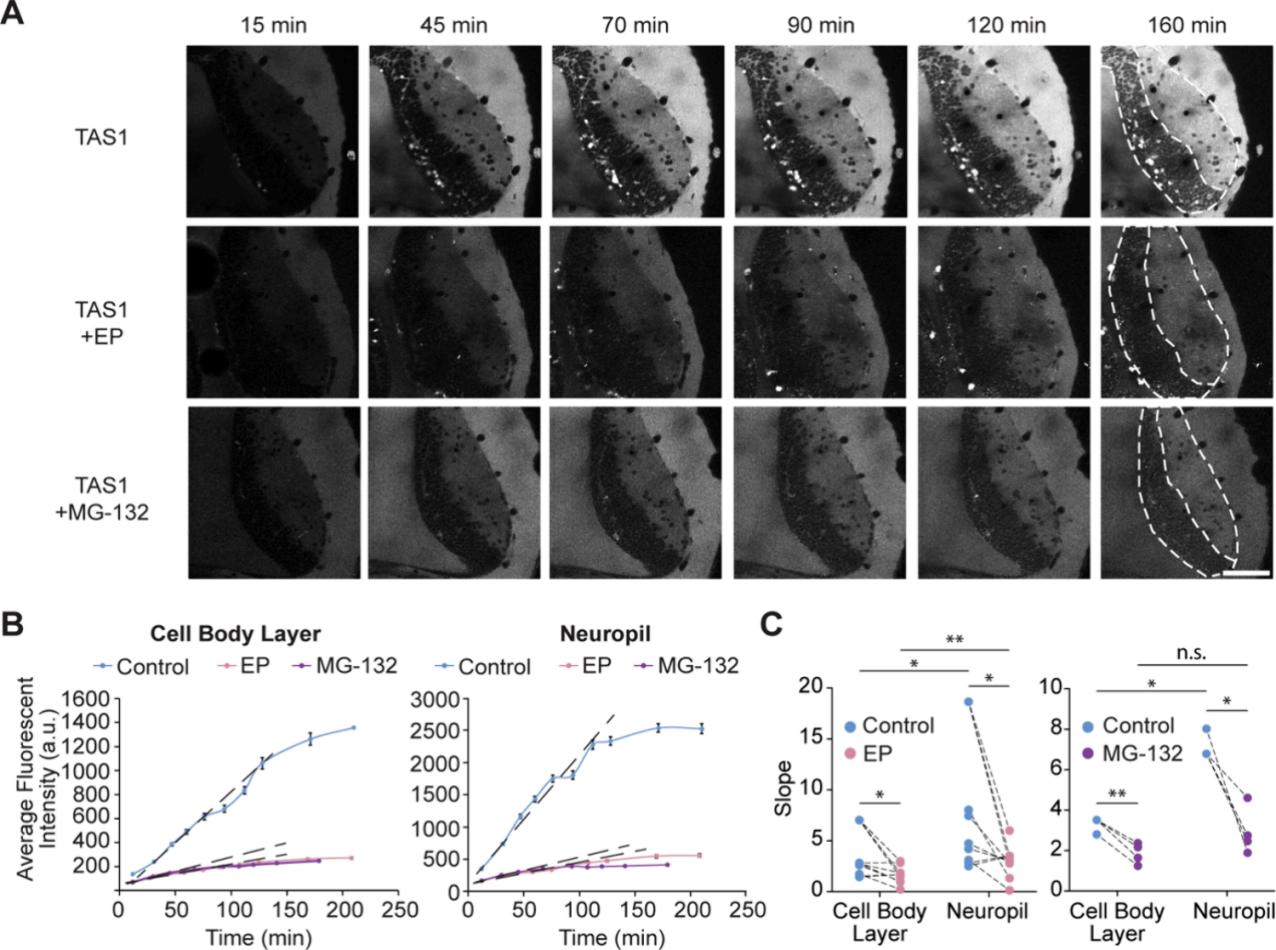
Epoxomicin (EP) and MG-132 effectively inhibit TAS1 measured proteolytic activity. (A) Representative time-lapse images of TAS1 fluorescent intensity across animals injected with TAS1 alone or coinjected with EP or MG-132. (B) Quantification of average fluorescence intensity over time in the cell body layer (CBL) and neuropil layer (N) in representative animals. The linear range of each curve was used to calculate the slope (dashed line). (C) Slope in CBL and N in control animals and those coinjected with proteasome inhibitors. Lines connect animals from the same batch imaged side by side. TAS1: *n* = 9, EP: *n* = 9, MG-132: *n* = 4; *: *p* < 0.05, **: *p* < 0.01, n.s.: not significant; two-way ANOVA with Holm–Šídák correction for multiple comparisons. Scale bar: 80 μm.

Proteasome activity has been shown to change in response to neuronal activity in primary neuronal cultures^13,14^ and tissue lysates.^15,16^ To determine whether the TAS1 probe is sensitive to changes in proteasome activity *in vivo*, we examined proteasome activity in different brain regions in response to pharmacologically increased neuronal activity. We preinjected animals with a GABA_A_ receptor antagonist, bicuculline (Bic), which blocks GABAergic inhibition and acutely increases neuronal activity in the tadpole brain.^6^ Similar pharmacological manipulations have been demonstrated to increase proteasome activity and recruit 26S proteasomes to neuronal dendrites in cultured neurons.^13,17^ We first confirmed the effect of Bic treatment on the overall proteasome activity in the tadpole brain using *in vitro* assays with Suc-LLVY-AMC. As expected, 25 minutes of Bic treatment significantly increased proteasome activity in tadpole brain lysates compared to vehicle-injected controls (Figure 5A). We then injected Bic-treated animals with TAS1 for time-lapse *in vivo* imaging (Figure 5B). Compared to vehicle-injected controls, Bic-treated animals showed a faster increase in TAS1 fluorescent signal across both the cell body layer and the neuropil layer, suggesting higher levels of overall proteasome activity in Bic-injected animals compared to batch-matched TAS1-only controls (Figure 5B–D). These results demonstrate that the TAS1 probe is sufficiently sensitive to detect changes in proteasome activity *in vivo*. Importantly, with TAS1-based *in vivo* imaging, we were able to observe a significantly higher magnitude of Bic-induced increase in proteasome activity in the neuropil layer compared to the cell body layer (Figure 5E). This subcellular difference was not distinguishable with the *in vitro* assay using brain lysates, which combined both the cell body and neuropil layers. This data corroborates prior reports in cell cultures^13,17^ and suggests that neuronal activity may differentially regulate proteasome activity across different subcellular compartments in the brain.

**Figure 5:**
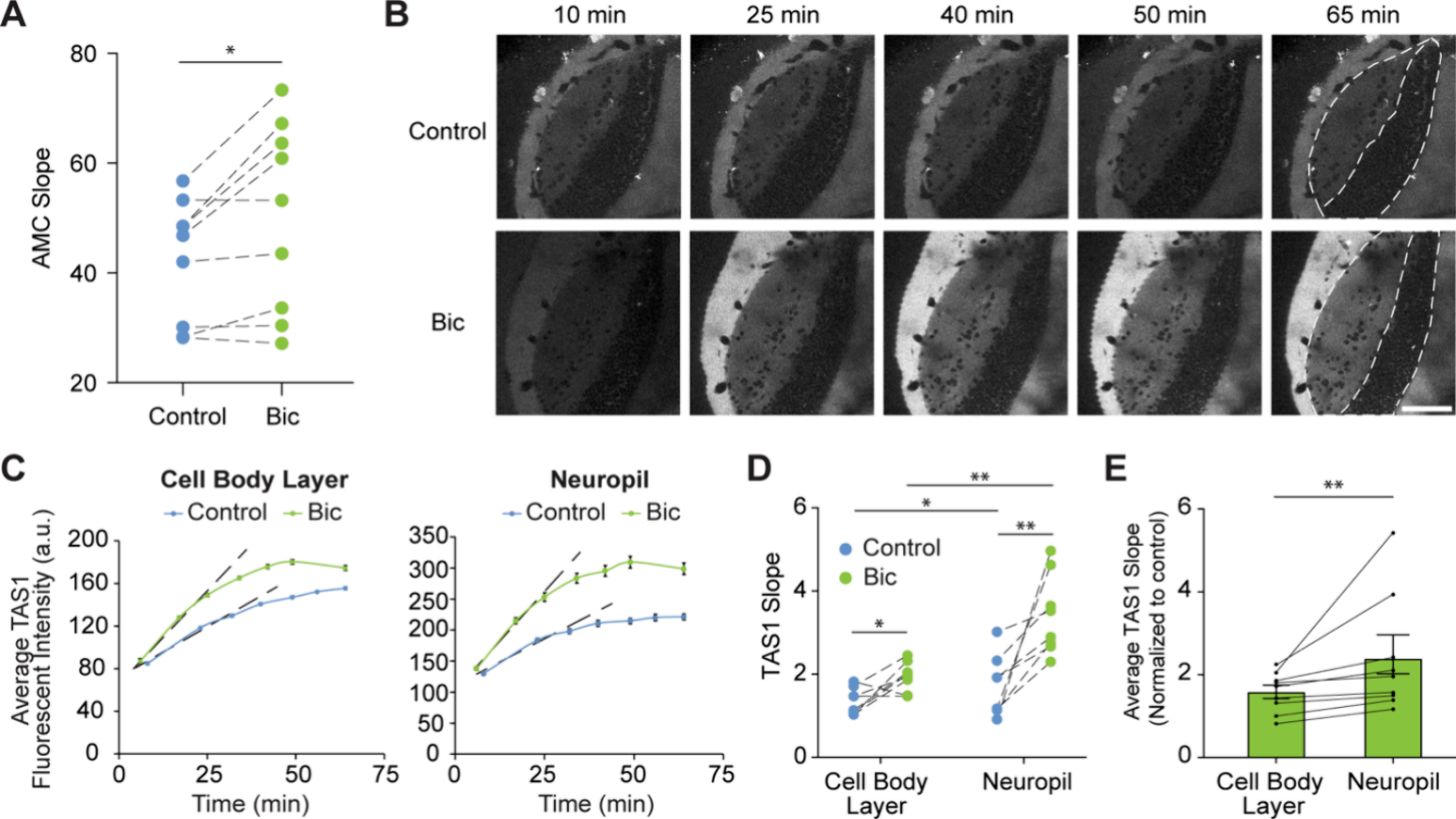
Bicuculline (Bic) increases proteasome activity in the tadpole brain. (A) Evaluation of proteasome activity in tadpole brain lysates using *in vitro* Suc-LLVY-AMC assay. (B). Representative time-lapse images of TAS1 fluorescent intensity in the optic tectum of control and Bic-treated animals. (C) Quantification of average fluorescent intensity over time in the cell body layer (CBL) and neuropil layer (N) in representative animals. The linear range of each curve was used to calculate the slope. (D) Slope in CBL and N in control animals and those exposed to Bic. Lines connect animals from the same batch imaged side by side. TAS1: *n* = 9, Bic: *n* = 9; *: *p* < 0.05, **: *p* < 0.01; two-way ANOVA with Holm–Šídák correction for multiple comparisons. (E) Average slope in CBL and N in the presence of Bic, normalized to batch-matched TAS1 only control. *n* = 9, **: *p* < 0.01, Wilcoxon matched-pairs signed rank test. Scale bar: 80 μm.

The temporal dynamics of the accumulation of TAS1 signal *in vivo* likely resulted from the net outcome of multiple processes: 1) The loading of the probes into cells; 2) The cleaving of the loaded probes inside cells (hence the production of fluorescence); and 3) The clearing of both uncleaved probes and cleaved fluorescent fragments from cells by normal metabolic processes. The signal we observed with the Atto590 dye suggests that loading of the probes into the cells is fairly rapid, which is consistent with prior observations and reports on the fast diffusion of membrane-permeable small molecules in the tadpole brain following intraventricular injection.^6^ Therefore, the level of fluorescence in cells reflects the net outcome of the total amount of probes loaded, the speed of enzymatic degradation (proteasome activity), and the speed of metabolic clearing. The plateau of the fluorescent signal we observed in the live brain under both basal and pharmacologically stimulated conditions likely resulted from the limited amount of probe injected and the relatively high level of proteasome activity in the intact brain, in addition to the clearing of both probes and cleaved fluorescent fragments. In the presence of proteasome inhibitors, the speed of cleaving was significantly reduced and lagged behind the speed of clearing, resulting in a much lower level of fluorescence intensity at the plateau. By keeping the amount of probe injected consistent across animals, we showed here that the slope of the initial linear range of the fluorescence accumulation curve can be used for quantitative evaluation of the overall proteasome activity in live tissue for both across- and within-animal comparisons.

The ability of TAS1 to detect changes in proteasome activity in live tadpole brains demonstrates its potential utility as a tool to measure proteasome-specific activity *in vivo* with enhanced spatial and temporal resolution. The higher proteasome activity in the neuropil compared to the cell body layer we observed under both basal and pharmacologically stimulated conditions is very intriguing. This observation likely reflects the need for local protein degradation at synapses to support important processes such as signal transmission and synaptic plasticity.^11,38^ The differential level of fluorescent TAS1 signal accumulated in the cell body layer versus the neuropil layer suggests that, contrary to the relatively fast and even loading of the probes, once the probes are taken up by neurons, their diffusion within the neuron is much more limited. Our results provide direct *in vivo* evidence for differentially upregulated proteasome activity across subcellular compartments in response to increased neuronal activity, extending prior observations from primary neuronal cultures^13,14,17–19^ and tissue lysates.^15,16^ Given the highly compartmentalized subcellular structures and functional regulation in neurons,^11^ the potential to directly monitor proteasome activity *in vivo* with subcellular resolution will greatly facilitate the search for compartment-specific regulatory mechanisms required for proteostatic control in neurons.

The TAS1 puncta localized to the lysosomes we observed in the tadpole brain at later time points during the time-lapse imaging could have resulted from two sources: either the TAS1 probe diffused into or was taken up by lysosomes and was subsequently cleaved in the lysosomes or the cleaved fluorescent peptoid-Rh110 fragment diffused into lysosomes and accumulated there. As the cleaved fluorescent peptoid-Rh110 fragment is uncharged (Figure 1A), it is possible that it functions similarly as LysoTracker Red and diffuses readily across membranes into acidic organelles in its uncharged form, becomes protonated, and gets trapped in these organelles. Other rhodamine dyes have also been previously reported to colocalize with LysoTracker.^39^ Since TAS1 puncta were mostly observed at the later time points during the time-lapse imaging and the TAS1 signal was reduced in the presence of proteasome inhibitors (Figure 3), we reason that these puncta are most likely a result of the cleaved probe entering the lysosomes by way of either diffusion or uptake and subsequently becoming protonated and thus trapped in these acidic organelles.

Overall, as a proof-of-principle study, our results highlight the promising applications of the TAS1 probe to study proteasome activity with enhanced spatial and temporal resolution in intact neural circuits by using *in vivo* time-lapse imaging. Future studies using such substrate-based fluorogenic probes with *in vivo* imaging techniques as well as further development of probes with faster temporal dynamics and specialized specificity will facilitate the investigation of the spatiotemporal dynamics of proteasome activity and its functional roles in the nervous system under both physiological and pathological conditions.

## Materials and Methods

### Animals

Albino *Xenopus laevis* embryos were obtained from in-house fertilization or Xen Express (Brooksville, FL) and reared at 21 to 22 °C with a 12-h dark/12-h light cycle in a 0.1× Steinberg solution (in millimolar: 58.0 NaCl, 0.67 KCl, 0.34 Ca­(NO_3_)_2_, 0.83 MgSO_4_, and 3.0 HEPES, pH 7.2). Animals were fed beginning at stage 47.^40^ All animal protocols were approved by the Institutional Animal Care and Use Committee (IACUC) of Georgetown University. Late stage 46 to 48 tadpoles of either sex were used for all experiments.

### *In Vivo* Two-Photon Imaging and Data Analysis

Animals were first staged based on gut morphology to ensure all the animals used in a single experiment were of the same developmental stage. Animals of approximately equal size were chosen for each experiment to ensure that brain size remained relatively constant across animals. Selected animals were immobilized with pancuronium dibromide (1 mM in 0.1× Steinberg solution) for 1 minute.^41^ Animals were then embedded in an imaging dish using agarose gel, and the imaging dish was filled with an oxygenated Steinberg solution. The health of the animal was monitored by the blood flow rate under the effect of pancuronium dibromide. Animals with significantly reduced blood flow were removed from the experiment. 40 nL of injection solutions with TAS1 (500 μM), Atto590, or Bic (100 μM) was injected intraventricularly per animal using a Picospritzer and calibrated microinjection capillaries. Proteasome inhibitors, EP (100 μM) and MG-132 (100 μM), were added to TAS1 injection solutions wherever applicable. All concentrations listed were for injection solutions. A higher concentration of reagents was used for *in vivo* injections compared to *in vitro* applications to account for the dilution in the ventricle (estimated to be ∼ 200–300 nL^6^) and the brain tissue. For animals treated with each experimental condition, at least one control animal from the same batch was imaged side by side.

A Bruker Ultima Investigator multiphoton microscope in resonant scanning mode with a 20× water immersion objective (Olympus XLUMPLFLN20XW, 1.0 NA) was used for two-photon imaging. A wavelength of 860 nm was used to excite the TAS1 probe. Image stacks from one lobe of the optic tectum were acquired at 2× magnification approximately every 10 to 20 minutes, with the total imaging duration ranging from 90 to 180 minutes. Each image stack spanned 100 μm with 2 μm steps between slices. Blood vessels on the dorsal side of the tectum and patterns of neuronal soma in the tectal lobe were used as references to ensure that the stacks were imaged at the same optical plane across time points. An example Z-stack video at 1× magnification is included in the Supporting Information.

Every fifth image in each 100 μm stack was chosen for data analysis. Time series of the tectal lobes at selected slices were imported into ImageJ (NIH) for analysis. The StackReg plugin was used to ensure alignment of the tectal lobe across time points. Three regions of interest (ROIs) were selected in both the cell body layer and neuropil layer. The cell body layer consists of a neuronal soma, and the neuropil layer consists mostly of axons and dendrites. The Time Series Analyzer (Version 3.0) plugin was used to obtain the average fluorescent intensity in each ROI across time points. Average fluorescent intensity was then averaged across selected slices and across all 3 ROIs in either the cell body layer or the neuropil. Average fluorescent intensity over time was plotted for the cell body layer and the neuropil, with 0 minutes defined as the time of TAS1 injection. The change in fluorescence intensity over time was quantified by taking the slope of the linear regression fitted on the linear range of the average fluorescence intensity curve over time. The linear range was determined by the most linear portion of the average fluorescent intensity curve (i.e., from the first time point to when the intensity starts to plateau) in control samples from each batch of experiments. For the same set of experiments, the same number of time points (≥3) was included in the linear range for the slope calculation. The linear regression was not forced to pass through zero. MATLAB (R2024a) was used for data processing and slope analysis.

### Colocalization Analysis

Animals were prepared for two-photon imaging, as described above. Animals were coinjected with 40 nL of TAS1 (500 μM) and LysoTracker Red (10 μM). Images were acquired 2 hours after injection at 4× magnification using a Bruker Ultima Investigator multiphoton microscope with a 20× water immersion objective (Olympus XLUMPLFLN20XW, 1.0 NA). A wavelength of 800 nm was used to excite both TAS1 and LysoTracker Red, as this wavelength resulted in the least amount of bleed-through between the TAS1 and LysoTracker channels. Animals injected with TAS1 only or LysoTracker Red only were used to confirm minimal bleed-through across the channels.

Acquired images were analyzed in ImageJ. Manual thresholding was performed in ImageJ to identify TAS1 and LysoTracker Red puncta. The Analyze Particles function in ImageJ was then used to define regions of interest (ROIs) based on TAS1 puncta. These TAS1 ROIs were overlaid onto the LysoTracker Red image, and TAS1 ROIs that overlapped with the LysoTracker Red puncta were visually identified. Percent colocalization was then calculated as the percentage of overlapping puncta out of the total number of TAS1 ROIs.

### In Vitro Proteasome Activity Assay

To evaluate the level of proteasome activity in tadpole brain tissue following pharmacological stimulation by bicuculine, tadpoles were intraventricularly injected with bicuculine (100 μM) 25 minutes before dissection. Whole brain samples from 6 to 8 animals were collected and pooled as one sample. Brain samples were homogenized in a lysis buffer containing HEPES (50 mM), EDTA (5 mM), NaCl_2_ (150 mM), Triton X-100 (1%), and glycerol (10%). The protein concentration in the lysate was determined by a BCA assay. AMC conjugated fluorogenic substrate Suc-LLVY-AMC (Medchem; cat.# HY-P1002) was used to quantify chymotrypsin-like (β5) proteasome activity in brain lysates. 4 μg total protein of lysates per sample was loaded in triplicate in 96-well plates with an activity assay buffer containing Tris-HCl (50 mM), KCl (40 mM), MgCl_2_ (5 mM), ATP (0.5 mM), DTT (1 mM), BSA (1 mM), and Suc-LLVY-AMC (10 μM). To control for non-proteasomal cleavage of substrates, a separate set of samples was plated with 10 μM of epoxomicin added to the reaction mixture. The fluorescent intensity was measured at 10 minute intervals for 2 hours using a microplate reader (BioTek SYNERGY H1; λ_ex_: 360 nm, λ_em_: 460 nm). The slope of the linear regression of the measured fluorescent intensity over time was calculated for each well. The average slope of the epoxomicin-treated samples was subtracted from the slope of the corresponding untreated samples to determine the overall proteasome activity for each sample. All bicuculline-treated samples were matched with nontreated controls from the same batch.

### Statistical Tests

Statistical tests were performed using GraphPad Prism version 10.5.0 for Mac OS (GraphPad Software, San Diego, California USA). The Shapiro–Wilk test for normality was used to verify normality of data sets. All bar graph data are presented as mean ± SEM. Data are considered significantly different when p values are less than 0.05. The statistical test used for each experiment is specified in the figure legends. All analyses were blinded to experimental conditions.

## Supplementary Material



## Notes

Representative movie showing a Z-stack from the dorsal surface to 150 μm of the tadpole brain (centered on the midbrain) imaged 45 minutes after TAS1 injection (MP4)
